# Constellations of Thought: Astrocytic Contributions to Cognition Across Rodent Models of Brain Dysfunction

**DOI:** 10.3390/biom16050662

**Published:** 2026-04-29

**Authors:** Konstantin Andrianov, Inna Gaisler-Salomon

**Affiliations:** 1 School of Psychological Sciences, University of Haifa, Haifa 3498838, Israel; igsalomon@psy.haifa.ac.il; 2 Integrative Brain and Behavior Research Center, University of Haifa, Haifa 3498838, Israel

**Keywords:** astrocyte, cognition, behavioral, GFAP, calcium signaling, gliotransmission, reactive astrogliosis, rodent models

## Abstract

Astrocytes are now recognized as active and essential participants in neural circuit function, extending far beyond their traditional roles as passive support cells. Emerging evidence highlights their critical involvement in synaptic modulation, information processing, and complex behaviors, making them key targets for understanding cognitive dysfunction in psychiatric disorders. This narrative review synthesizes current findings from rodent models to elucidate the relationship between astrocytic networks and multidomain cognitive performance. We first outline the morphological and physiological features of astrocytes, followed by a comprehensive overview of the modern experimental toolkit, including observational markers and advanced interventional strategies. Next, we evaluate commonly used behavioral assays that capture distinct cognitive domains, ranging from basic spatial and recognition memory to higher-order executive functions, cognitive flexibility, and social cognition. By integrating recent experimental evidence, we detail the specific mechanistic pathways, such as intracellular calcium signaling, gliotransmission, and neuroinflammatory reactivity, through which astrocytes directly govern these cognitive processes. Finally, we highlight critical knowledge gaps stemming from methodological limitations, arguing for the integration of more ethologically relevant, high-throughput behavioral tasks alongside highly specific targeting tools to better capture the functional heterogeneity of astrocytes in cognitive health and disease.

## 1. Introduction

Astrocytes are a distinct type of glial cells within neural tissue. Initially considered passive support cells responsible for maintaining homeostasis and providing metabolic and structural support to neurons, astrocytes are now recognized as central players in CNS function, with distinct molecular, morphological, and functional profiles across brain regions. A growing body of research demonstrates that astrocytes actively participate in synaptic modulation, neurotransmitter regulation, and signal integration, thereby directly influencing neuronal communication and information transfer in the central nervous system (CNS). This review will focus on the role played by CNS astrocytes in cognitive dysfunction. We first outline the known morphology and physiology of CNS astrocytes, focusing on features that may contribute to learning, memory, and attention. Next, we summarize current experimental approaches for investigating astrocyte function and cognition in animal models. Finally, we discuss evidence from rodent studies that highlights astrocytic involvement in specific cognitive domains relevant to neurological and psychiatric illnesses.

## 2. CNS Astrocytes: Morphology and Physiology

Astrocytes are characterized by their star-like shape, from which their name is derived. Based on their morphological appearance and regional distribution, astrocytes were traditionally classified into two categories: protoplasmic (Type 1) astrocytes, which have multiple truncal branches that divide into numerous, finely branched projections and are found predominantly in gray matter, and fibrous (Type 2) astrocytes, which have a long, filamentous neuron-like appearance and are found mostly in white matter [1,2,3]. In addition, specialized astrocyte variants such as Bergmann glia and Müller cells exhibit region-specific roles [4]. Astrocytes in different regions differ in morphology, surface cell marker expression, and chemokine and cytokine production [5]. Several astrocyte variants can coexist within a single brain region [6]. Importantly, morphological classifications do not fully capture astrocyte diversity, as these cells also exhibit pronounced functional heterogeneity in their signaling properties, metabolic roles, and interactions with neuronal circuits across brain regions and physiological states [6].

Traditionally, astrocytes were regarded as supportive cells that provided structural and metabolic maintenance for neurons. They physically enwrap neuronal somata and synapses, supplying nutrients, regulating ion balance, and maintaining the extracellular environment [7]. Beyond these fundamental roles, astrocytes are now known to engage in specific and dynamic interactions with neurons that distinguish them from other glial cells. They exhibit finely regulated fluctuations in intracellular calcium concentrations in response to neurotransmitter release. These calcium transients can propagate through astrocytic networks as calcium waves, triggering the release of signaling molecules known as gliotransmitters, which in turn modulate neuronal excitability and synaptic plasticity [8].

Gliotransmitters released by astrocytes include glutamate, ATP, GABA, and D-serine [7,9]. Through these messengers, astrocytes influence both excitatory and inhibitory transmission. They modulate synapses not only by direct perisynaptic contact but also through secretion of soluble factors that shape synaptic strength and structure [10]. A single astrocyte can enwrap multiple neuronal somata and contact between 300 and 600 dendrites within its domain [11]. This extensive morphological reach enables each astrocyte to influence hundreds of synapses simultaneously, coordinating neuronal activity and contributing to network-level synchronization across circuits [12]. Astrocytes also form extensive intercellular networks through connexin proteins, primarily connexin 43 and connexin 30, which assemble into gap junctions and hemichannels. While gap junctions enable the exchange of ions and metabolites between astrocytes under physiological conditions, hemichannels are typically closed but can open in response to stress or inflammatory signals, leading to the release of gliotransmitters such as ATP and glutamate that can indirectly modulate neuronal activity and synaptic function. 

These insights have given rise to the concept of the tripartite synapse, in which a pre-synaptic neuron, a post-synaptic neuron, and an astrocyte form a functional synaptic unit that enhances synaptic diversity and communication [13]. Astrocytes express a broad array of neurotransmitter receptors, including AMPA and NMDA glutamate receptors, metabotropic glutamate receptors, GABA receptors, and D2 dopamine receptors [12]. This diversity allows astrocytes to detect and respond to a wide range of synaptic signals. Moreover, certain receptor and transporter subtypes are uniquely localized to astrocytic membranes within the tripartite synapse. For example, the mostly astrocytic glutamate transporters, glutamate–aspartate transporter (GLAST; also known as EAAT1) and glutamate transporter-1 (GLT-1; also known as EAAT2), are the principal transporters responsible for clearing synaptic glutamate and maintaining optimal extracellular glutamate levels [14,15]. Another example of a molecule that plays an important role in astrocyte-neuron glutamate dynamics is the metabolic enzyme glutamate dehydrogenase 1 (GDH, *Glud1*), which catabolizes the breakdown of glutamate to alpha-ketoglutarate, linking glutamate transmission to energy metabolism predominantly in astrocytes [16,17].

From a developmental perspective, astrocytes are crucial for neural circuit formation. They regulate synapse formation, maturation, and function through both contact-mediated and secreted signals [18]. Neuronal synapse formation begins only after astrocytes have formed, further emphasizing their critical role in synaptic development [19]. Once formed, the tripartite synapse represents a locus of bidirectional communication between neurons and astrocytes, allowing glial cells to modulate the induction and maintenance of synaptic plasticity, long-term potentiation (LTP), and other processes fundamental to learning and cognition.

Beyond their direct interactions with neurons, astrocytes play central roles in several mechanisms that indirectly affect neuronal stability and function [20]. For example, astrocytes are central to neurovascular coupling, contributing to blood vessel constriction and dilation and to the supply of oxygen and glucose to metabolically active brain regions. Furthermore, astrocytes contribute to brain barrier maintenance, particularly during states of injury. Astrocytes also actively participate in neuroinflammatory signaling by sensing injury- or stress-related cues and responding through the release of cytokines, chemokines, and other inflammatory mediators. In turn, they can amplify or modulate immune responses by interacting with microglia and influencing blood–brain barrier permeability, thereby regulating the recruitment of peripheral immune cells. Additionally, inflammatory signaling can alter astrocytic functions such as neurotransmitter uptake and gliotransmission, further impacting neuronal activity and circuit function [21]. Injury or stress exposure can also lead to reactive astrogliosis, a process characterized by hypertrophy, upregulation of GFAP, and, in some cases, scar formation, which can further disrupt neuronal signaling and information flow [12,22,23].

Emerging evidence suggests that astrocytes may also exhibit sex-dependent differences in morphology, gene expression, and functional responses to neuromodulatory and inflammatory signals, often in a region- and hormone-dependent manner. For example, sex differences have been reported in astrocyte density and complexity in specific brain regions, as well as in calcium signaling and inflammatory reactivity [24,25].

The mechanisms outlined above can contribute to cognitive dysfunction across multiple neurological and psychiatric disorders. Major categories of psychiatric disorders include mood disorders such as major depressive disorder and bipolar disorder, anxiety and trauma-related disorders, schizophrenia spectrum and other psychotic disorders, and neurodevelopmental disorders including autism spectrum disorder and attention-deficit/hyperactivity disorder. Across these conditions, astrocytic dysfunction has been linked to specific pathophysiological processes, including impaired glutamate uptake and potassium buffering, altered gliotransmission, disrupted neurovascular coupling and metabolic support, and aberrant neuroinflammatory signaling. These alterations can, in turn, differentially impact cognitive domains such as attention, executive function, and memory, suggesting that distinct astrocytic mechanisms may contribute to disorder-specific cognitive profiles [26,27].

## 3. Studying Astrocytes: Experimental Approaches

**Figure 1:**
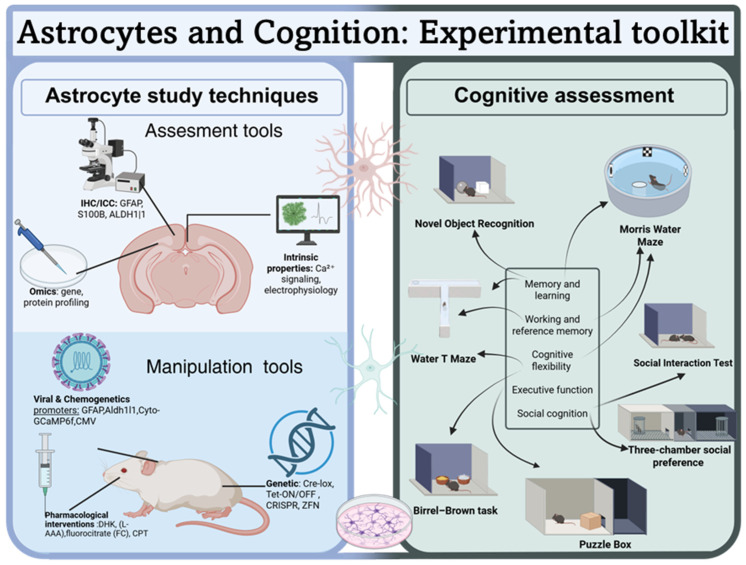
Biochemical and behavioral tools used to study astrocytes function in cognition.

Several tools are used to visualize changes in astrocytic structure or function, or for manipulating astrocytes directly (see Figure 1). In the present review, we focus on the Glial Fibrillary Acidic Protein (GFAP), S100B, and 10-formyltetrahydrofolate dehydrogenase (Aldh1l1) due to their widespread use in studies linking astrocytes to cognitive function. Other markers, e.g., N-Myc downstream-regulated gene 2 (NDRG2), GLAST, and GLT-1, are used to label astrocytic populations and have been described elsewhere [3].

Glial Fibrillary Acidic Protein (GFAP) is an intermediate filament protein that provides astrocytes with structural stability and supports key processes involved in repair, plasticity, and reactive gliosis [28]. While predominantly expressed in astrocytes and commonly used to distinguish astrocytes from neurons, GFAP is not expressed uniformly across all astrocyte populations. Thus, GFAP is abundantly expressed in fibrous astrocytes of the white matter and hippocampus but limited in protoplasmic astrocytes within the healthy cerebral cortex [29,30]. GFAP is also expressed in neural stem cells located in neurogenic regions, which reduces its specificity [7]. Multiple GFAP isoforms, including α, β, γ, δ, and κ, show region- and state-dependent expression, but their individual functions remain poorly understood. Notably, GFAP upregulation is a hallmark of reactive astrogliosis and is driven by a range of signaling pathways activated under pathological conditions, including cytokine-mediated signaling (e.g., via IL-1β, TNF-α, and JAK/STAT pathways), as well as injury- and stress-related cues [21]. While a detailed discussion of the molecular regulation of astrocyte reactivity is beyond the scope of this review, these pathways are important as they shape astrocytic functional states that can ultimately influence neuronal activity. Despite its limitations, GFAP continues to serve as a practical and informative tool, particularly for detecting reactive gliosis and white matter astrocytes, and often in combination with complementary astrocyte markers [31,32].

S100B is a cytosolic calcium-binding protein abundantly expressed in mature CNS astrocytes, where it plays a key role in calcium signaling, cytoskeletal regulation, and neurotrophic support. Notably, S100B is expressed to a lower degree in oligodendrocytes, ependymal cells, the choroid plexus epithelium, vascular endothelial cells, lymphocytes, and several neuronal populations, particularly during development [33]. Moreover, S100B expression is regionally variable: in the adult rodent and human brain, S100B-positive astrocytes are particularly abundant in the hippocampal formation, corpus callosum, and subcortical white matter, but significantly less common in cortical gray matter [34,35]. Similarly to GFAP, S100B expression increases markedly during reactive astrogliosis, making it a reliable indicator of astrocyte activation in pathological contexts that involve neuroinflammation [34]. In summary, S100B is a robust but non-exclusive astrocytic marker that reflects both astrocyte abundance and activation state.

Aldh1l1 is a cytosolic enzyme involved in folate metabolism. In CNS, it is abundantly and uniformly expressed in astrocytes, with a substantially broader pattern of astrocyte expression than GFAP and S100B [36]. Under normal physiological conditions, it shows minimal neuronal expression, although limited off-target expression has been reported, e.g., in immune cells [31,37,38]. Aldh1l1 is expressed in both gray and white matter astrocytes starting in early development, and its expression seems to be stable across resting and reactive states [36].

Astrocytic type specificity, regional distribution, and developmental considerations should guide the choice of suitable astrocytic markers. While all three markers can be used to visualize astrocytic features or serve as promoters to drive astrocyte-targeted expression in viral vectors and transgenic strategies (see below), their limitations should be considered for each specific study.

### 3.1. Visualizing Astrocytes In Vitro and In Vivo

Astrocyte markers are used to characterize astrocyte morphology and assess changes in response to experimental manipulations. The most common method to visualize astrocytes is immunocytochemistry or immunohistochemistry. Astrocytes immunolabeled with GFAP, S100B, Aldh1l1, or other markers can be examined for changes in cell density, morphology, or co-expression with other cellular markers. In addition to structural analysis, intrinsic properties of astrocytes can be investigated both in vitro and in vivo. For instance, Ca^2+^ signaling dynamics or electrophysiological properties can be assessed in cultured astrocytes, acute brain slices, or living animals using two-photon microscopy [39,40,41]. Gliotransmitter release can be studied using sniffer patch recordings, a technique in which a membrane patch enriched with receptors (e.g., NMDA) is positioned near an astrocyte to detect released signaling molecules [42]. Finally, astrocytes can be isolated and purified from mixed cell populations using methods such as fluorescence-activated cell sorting (FACS) or magnetic-activated cell sorting (MACS), and subsequently cultured for molecular or transcriptomic analyses [43].

### 3.2. Manipulating Astrocyte Function

Several methodological approaches can be taken to manipulate astrocyte function. Pharmacological tools developed to impact astrocytic function include dihydrokainic acid (DHK), a selective inhibitor of the GLT-1 glutamate transporter used to disrupt astrocytic glutamate uptake [44]; L-α-aminoadipate (L-AAA), an astrocyte-selective exotoxin that induces reversible astrocytic damage and is used to assess the consequences of astrocyte ablation [45,46]; and fluorocitrate, which preferentially accumulates in astrocytes and inhibits the glial tricarboxylic acid (TCA) cycle, thereby reducing astrocytic metabolic activity [47]. The major limitation of pharmacological approaches lies in their lack of cellular specificity and potential off-target effects, making it challenging to isolate astrocyte-specific contributions without influencing neuronal or systemic processes [47,48].

Strategies that involve viral vectors engineered with astrocyte-selective promoters are used to selectively target astrocytic populations. Promoters such as GFAP or its shortened derivatives (the gfa2 fragment and GfaABC1D) can be incorporated into these vectors to enable transgene expression or conditional gene deletion specifically within astrocytic populations [49]. Such strategies can result in gene deletion, under-expression, or over-expression, enabling targeted investigation of astrocyte-specific molecular mechanisms. For example, astrocyte-specific knock-in expression of genetically encoded calcium indicators, such as Cyto-GCaMP6f, enables visualization of intrinsic astrocytic calcium dynamics in vivo [50]. Such tools also allow for controlled and reversible modulation of astrocyte activity, using chemogenetic receptors such as hM3Dq or promoter-driven tetanus neurotoxin (TeNT) expression to suppress vesicular release [51,52,53]. Alternatively, constitutive gene knock-in strategies can be applied to study the long-term effects of stable genetic alterations on astrocyte physiology, astrocyte-neuron interactions, and behavior. An important consideration when interpreting GFAP-driven strategies is that promoter activity can vary across brain regions and may be altered under pathological or reactive conditions, potentially affecting targeting specificity and expression levels in both viral and transgenic models.

Genetically modified mice are used to investigate astrocytic involvement in various phenotypes across development. Several astrocyte-specific Cre recombinase mouse lines are available and have been used either with floxed (loxP-flanked) mice or other recombinase-responsive mice [54]. The Tet-ON/OFF system provides temporal control of astrocyte-specific gene expression [55]. For example, Aldh1l1-creER mice have been used to selectively delete genes of interest in adult astrocytes, thereby bypassing developmental effects. Furthermore, some studies have employed a combinatorial approach, leveraging both the GFAP-creER and GFAP-tTA systems to achieve temporal control and on/off regulation of astrocyte-specific gene expression [52]. CRISPR/Cas9 and zinc-finger nucleases (ZFNs) have also been used to selectively manipulate genes of interest in astrocytes [56,57].

## 4. Rodent Models of Cognitive Function

Cognitive deficits are a central feature of several psychiatric and neurological disorders, and include a decline in attention, memory, and executive capacities. Specifically, this review focuses on deficits in episodic and working memory, executive function, cognitive flexibility, and social cognition [58,59].

Animal studies are essential for investigating the neurobiological substrates of cognition because they enable cell-type-specific manipulations and direct assessment of causal relationships among cellular dysfunction, circuit-level changes, and cognitive behavior. These approaches are not feasible in humans and cannot be adequately captured in vitro. Bridging the gap between human cognition studies and animal models remains complex, as many rodent cognitive tasks require extensive training, specialized equipment, and labor-intensive procedures, and are implemented heterogeneously across labs. Nevertheless, they remain a central tool for studying the neural substrates of cognition, particularly the role of astrocytes. 

In this review, we focus on visual and spatial memory, cognitive flexibility, executive function, and social cognition. We outline several behavioral assays, ranging from commonly used, well-validated paradigms to recently introduced techniques that capture distinct cognitive domains associated with psychopathology. We prioritize tasks with high face validity and throughput, often relying on naturally occurring behaviors (see Figure 1). Performance on these tasks depends on neural circuits essential to human cognition, particularly the hippocampus and prefrontal cortex. We then review recent experimental evidence highlighting how astrocytic function may contribute to cognitive performance in specific behavioral tasks. Individual studies on astrocyte control of cognitive capacities are summarized in Table 1. The studies included in the Table (and in Section 5 below) were selected according to (i) the cognitive task (MWM, NOR, Puzzle Box Task, Birrel-Brown, or Water T-maze to assess cognitive flexibility) and (ii) astrocytic manipulation. Studies assessing the impact of environmental manipulation on cognitive function via changes in astrocytic function were also included.

Notably, several paradigms affected by astrocyte-specific manipulations were not included in this review, as we aimed to focus on high-throughput tasks suitable for combined behavioral batteries. For example, dry-land mazes (e.g., Y-maze, T-maze), although commonly used to assess spatial working memory and cognitive flexibility, are limited by requirements such as food deprivation and relatively slow learning curves (e.g., [60]). Moreover, variations in the execution of these tasks, and behavioral tasks in general, may affect the comparability of findings.

**Table 1:** Detailed overview of studies on astrocyte control of cognitive function.

Study	Animal	Experimental Manipulation	Tools Used to Manipulate * or Visualize ** Astrocytes	Brain Region ***	Behavioral Test	Behavioral Impact of Manipulation
General or environmentally induced alteration of astrocyte function						
[61]	Rats (Sprague-Dawley)	Lesion of astrocytes	Pharmacological: La-aminoadipate (L-AAA) Chemogenetic: hM3Dq (GFAP) IHC: GFAP	IL-PFC	NOR	Impaired recognition memory
		Activation of astrocytes				Improved recognition memory
[62]	Rats (Sprague-Dawley)	Lesion of astrocytes	Pharmacological: La-aminoadipate (L-AAA) ***- Chemogenetic: hM3Dq (GFAP)	mPFC	ASST	Impaired cognitive flexibility
		Activation of astrocytes				Improved cognitive flexibility
		Infusion of S100β				Improved cognitive flexibility
[63]	Rats (Wistar-Han)	Lesion of astrocytes	Pharmacological: La-aminoadipate (L-AAA) IHC: GFAP	mPFC	ASST MWM	Impaired cognitive flexibility and reversal learning
[64]	Mice (C57BL/6J)	Whole-brain Astrocyte-*Cpt1a* knockout	Transgenic mice: Cpt1a ^lox/lox^ Viral: AAVgfaABC_1_D-Cre-GFP, AdV-CMV-Cre-GFP Astrocyte cultures Western Blot: GFAP	Whole brain	NOR	Impaired recognition memory
		GABAA receptor antagonist				Restored recognition memory
[65]	Mice (C57BL/6J)	Social isolation	IHC: GFAP		NOR	Impaired recognition memory
		Dihydromyricetin treatment				Restored recognition memory
[66]	Rats (Sprague-Dawley)	Running	IHC: GFAP, S100β		NOR ASST	Enhanced cognitive flexibility; No effect on recognition memory
[67]	Mice (Swiss)	Aging	IHC: GFAP		NOR MWM	Impaired recognition memory and spatial memory
		Environmental enrichment (EE)				Prevention of recognition memory and spatial memory impairments
[68]	Mice (Swiss)	Aging	IHC: GFAP 3-D reconstructions		NOR	Impaired recognition memory
		Environmental enrichment (EE)				Prevention of recognition memory impairments
[69]	Rats (Fischer F344)	Aging	Viral: Flap-CMV-GDNF-WPRE IHC: GFAP	CA1	MWM	Impaired spatial learning and memory
		Astrocytic GDNF overexpression				Restored spatial learning and memory
[70,71]	Rats (Wistar)	MWM Exposure	Imaging: PTAH staining	CA1 DG	-	-
Calcium Signaling & Homeostasis						
[56]	Mice (C57BL/6)	Astrocyte-specific deletion of Inositol 1,4,5-trisphosphate receptor, type 2 (ITPR2)	Transgenic mice: Aldh1l1/CreER; IP3R2 ^flx/flx^ Viral AAV-GFAP-IP3R2 shRNA Gene editing: CRISPR/Cas9	Whole brain mPFC	NOR SP	Reduced sociability Altered social novelty preference Enhanced recognition memory
[72]	Mice (C57BL/6)	Astrocyte-specific deletion of Inositol 1,4,5-trisphosphate receptor, type 2 (ITPR2)	Transgenic mice: GFAP/Cre; IP3R2 ^flx/flx^ Ca^2+^ imaging: SR101 IHC: GFAP	Whole brain	MWM	Intact spatial learning, memory and flexibility
[73]	Mice (C57BL/6)	Astrocytic calcium pump plasma membrane (PMCA2) over-expression	Viral: AAV-GfaABC1D-PMCA2 Chemogenetic: hM3Dq (GfaABC1D) Ca^2+^ imaging: CytoGCaMP6f (GfaABC1D) IHC: S100β	Cortex (bilateral)	SP SIT	Deficits in social interaction and preference
		Activation of astrocytes				Restored social interaction and preference deficits
[74]	Mice (C57BL/6)	Aβ injection	Transgenic mice: GFAP-CreERT2/CaNB11 ^flx/flx^ IHC: GFAP Western Blot: GFAP	Whole brain	NOR	Impaired recognition memory
		Astrocytic calcineurin B1deletion				Prevented recognition memory impairment
Gliotransmission & Synaptic Regulation						
[55]	Mice (C57BL/6)	Inhibition of vesicular release (dnSNARE)	Transgenic mice: GFAP-tTA/SNARE VAMP2/synaptobrevin II tetO IHC: GFAP, S100β	Whole brain	NOR MWM	Impaired recognition and spatial memory
		d-Serine administration				Restored recognition and spatial memory
[52]	Mice (C57BL/6)	Expression of tetanus neurotoxin (TeNT) in astrocytes	Transgenic mice: GFAP-tTA/TRE-TeNT Viral: lenti-hGFAP-TeNTΔ1-GFP Ca^2+^ imaging: (Fura-2 AM) IHC: GFAP	Whole brain	NOR	Impaired recognition memory
[75]	Mice (C57BL/6)	Astrocytic *Bmal1*deletion	Transgenic mice: *Glast* -CreER ^T2^/*Bmal1* ^flx/fx^ Astrocyte cultures IHC: GFAP Western Blot: GFAP RT–PCR	Whole brain	NOR	Impaired short-and long-term recognition memory
[76]	Mice (C57BL/6)	Anesthesia	Astrocyte cultures		NOR PBT	Impaired recognition memory and impaired problem solving
		Dexmedetomidine treatment				Restored recognition memory and problem solving
[77]	Mice (Swiss Kunming)	GLT-1 inhibition	Pharmacological: Dihydrokainic acid (DHK)	Lateral ventricles	NOR	Impaired recognition memory
[78]	Rats (FSL)	FSL rats d-Serine administration	IHC: GFAP Western Blot: GFAP, GLAST and GLT-1		NOR	Impaired recognition memory Restored recognition memory
[79]	Mice (C57BL/6)	GLAST deletion	Transgenic mice: GLAST KO	Whole brain	NOR	Deficits in social preference and recognition
[80,81]	Mice (C57BL/6)	*Glud1* deletion	Transgenic mice: Cre/Glud1 ^flx/flx^	Whole brain	NOR SP&R WTM	Impaired recognition memory: Deficits in social preference and recognition Impaired cognitive flexibility
[82]	Mice (C57BL/6)	*Glud1* partial deletion + mild stress	Transgenic mice: Cre/Glud1 ^flx/flx^	Whole brain	SP WTM	Intact social preference Impaired learning and cognitive flexibility
[83]	Rats (Sprague-Dawley)	Genetic disruption of system xc-mediated glutamate release from astrocytes	Gene editing: Zinc-finger nucleases (ZFNs) for Slc7a11 gen Astrocyte cultures	Whole brain	NOR ASST	Impaired cognitive flexibility Intact recognition memory
[84]	Mice	*Best1* deletion	Viral: AAV-GFAP-Best1-IRES-EGFP, AAV-GFAP104-GRABNE2m Astrocyte cultures IHC: GFAP Ca^2+^ imaging: jRCaMP1a(GFAP104)	CA1	MWM	Impaired cognitive flexibility
		Astrocytic re-expression of *Best1*				Rescued cognitive flexibility deficits
		d-Serine administration				Rescued cognitive flexibility deficits
[85]	Mice (C57BL/6)	Expression of mutant *DISC1* in astrocytes	Transgenic mice: GFAP-tTA/TRE-mutant DISC1	DG	NOR SP	Impaired social preference, Intact NOR
		d-Serine administration				Restored social preference deficits
[86]	Rats (Wistar)	Amyloid-beta (Aβ) oligomers injections	Viral: pAAV2/9-GfaABC1D-EGFP-3FLAGmicro30shRNA (Grin2a) IHC: GFAP	DG	NOR	Impaired recognition memory
		Knockdown of astrocytic *Grin2a*				Aggravated recognition memory deficits
[87]	Mice	Astrocytic Apolipoprotein E (ApoE) deletion	Transgenic mice: Aldh1l1-CreERT2/ApoE ^flx/flx^	Whole brain	MWM	Impaired spatial memory
		Controlled cortical impact (CCI)				Aggravated spatial memory deficits
[88]	Mice	Sleep deprivation	Transgenic mice: GFAP-tTA/tetO-dnSNARE Pharmacological: A1 receptor antagonist CPT Astrocyte cultures IHC: GFAP Ca^2+^ imaging: in situ (Rho d-2)	Whole brain	NOR	Impaired recognition memory
		Inhibition of vesicular release (dnSNARE)				Restored recognition memory
		A1 receptor antagonist CPT				Restored recognition memory
Inflammation & Reactivity						
[89]	Mice (ICR)	Lipopolysaccharide (LPS) injection	IHC: GFAP		MWM	Impaired spatial memory
		Resveratrol				Attenuated spatial memory impairment
[90]	Mice (C57BL/6)	Lipopolysaccharide (LPS) injection	Pharmacological: Fluorocitrate Astrocyte cultures RT-PCR	Lateral ventricle	MWM	Impaired spatial learning and memory
		IL-10 injection				Attenuated learning and memory impairment
		Metabolic inhibition of astrocytes				Attenuated learning and memory impairment
[91]	Rats (Sprague-Dawley)	Middle cerebral artery occlusion and reperfusion	Pharmacological: Fluorocitrate IHC: GFAP Western Blot: GFAP	Lateral ventricles	MWM	Impaired spatial learning and memory
		Metabolic inhibition of astrocytes				Alleviated spatial learning and memory impairment
[92]	Mice (C57BL/6)	Aged mice + surgery (hepatectomy)	Western Blot: GFAP IHC: GFAP		MWM	Impaired spatial learning and memory
		Minocycline treatment				Reversed spatial learning and memory impairment
[93]	Mice (C57BL/6)	Systemic ketamine			NOR	Impaired recognition Memory
[94]	Mice (C57BL/6)	Chronic kidney disease (CKD) by two-step 5/6 nephrectomy	IHC: GFAP		MWM	Impaired spatial learning and memory
		AST-120				Restored spatial learning and memory impairment
		NLRP3-knockout				Prevented spatial learning and memory impairment
[95]	Mice (C57BL/6)	Injections of cisplatin	Transgenic mice: GFAP-Cre/S1pr1 ^flx/fl^	Whole brain	PBT	Impaired problem solving
		Astrocyte- S1pr1 knockout				-
		Injections of FTY720 and ozanimod				Reversed problem-solving deficits
[96]	Mice (C57BL/6)	Carotid stenosis	Transgenic mice: Aldh1l1-Cre/Trpa1 ^flx/flx^ IHC: GFAP	Whole brain	NOR	Impaired recognition memory
		Astrocytic *TRPA1* deletion				Aggravated recognition memory deficits
[97]	Mice	Astrocyte deletion of R-like ER kinase (PERK).	Transgenic mice: GFAP-Cre/PERK ^fl/fl^ Astrocyte cultures IHC: GFAP, GLAST Western Blot: GFAP	Whole brain	MWM	Does not alter memory or cognitive flexibility
		Experimental stroke				PERK deletion aggravated memory and cognitive flexibility impairment
		Aging				PERK deletion aggravated memory and cognitive flexibility impairment

* Specific tools used to manipulate function of astrocytes as described in Section 3. Viral: specific viral vectors used to manipulate astrocytic function, including promoter and target gene. Transgenic: Promoter and recombinase systems with target gene. Chemogenetic: receptor and promoter used to manipulate astrocytes. Pharmacological agents ** Specific tools used to visualize or observe changes in astrocytic function, including promoters used for immunoimaging (IHC) or Western blot, calcium imaging dyes and genetic indicators with promoter, use of astrocytic cultures and RT-PCR. *** Brain region of astrocytic manipulation: whole brain, prefrontal cortex (mPFC), Hippocampus (CA1 and dentate gyrus (DG)). NOR: Novel object recognition. ASST: Attentional Set Shifting Task. MWM: Morris Water Maze. SP: Social preference, SP&R: Social preference and recognition. SIT: Social Interaction Test. PBT: Puzzle Box Task.

### 4.1. Visual and Spatial Memory

A commonly used assay for assessing visual and recognition deficits is the Novel Object Recognition (NOR) task. Originally described by Ennaceur and Delacour (1988) [98], this task is based on the spontaneous discrimination paradigm and typically involves a familiarization phase followed by a test phase, where the animal’s ability to recognize a previously encountered object is measured by the time spent exploring a novel object versus a familiar one. NOR leverages rodents’ innate preference for novelty without requiring external reinforcement or extensive training [99]. Moreover, the NOR task is considered a low-stress assessment that can be adapted to evaluate visual memory and recognition after short or long time spans, depending on the inter-trial interval (ITI) between the familiarization and test phases. A key limitation of this task is that performance can be strongly influenced by factors unrelated to recognition memory, including anxiety-like behavior, locomotor activity, and innate object preference.

Complementing the visual and recognition memory assessed by the NOR task, the Morris water maze (MWM) was developed to investigate spatial learning and memory in rats. MWM is frequently used to validate rodent models of neurocognitive disorders and to evaluate pharmacological treatments [100]. In a typical MWM protocol, rats or mice are placed in a water-filled tank with a submerged escape platform. Animals begin at various starting points in the tank, and the task is facilitated by spatial cues affixed to the tank walls that aid navigation toward the submerged platform [101]. One advantage of this paradigm is that animals’ innate aversion to water facilitates protocol execution without additional motivational training. Moreover, the MWM compels animals to rely solely on visual landmarks within the environment, excluding the use of other senses [102]. Furthermore, the task can be adapted to measure additional cognitive capacities, e.g., cognitive flexibility, by varying the platform location after initial task acquisition [84,87]. However, it should be kept in mind that escape latency can be confounded by stress reactivity, visual or motor impairments, and differences in swimming ability, which may obscure the interpretation of spatial learning and memory deficits.

### 4.2. Cognitive Flexibility and Attentional Set-Shifting

Cognitive flexibility plays a crucial role in enabling individuals to engage in complex tasks, including multitasking and finding adaptable solutions to changing demands [103]. A key aspect of cognitive flexibility is attentional set shifting- the ability to quickly switch focus between different rules, stimuli, or “mental sets” to adapt to changing environments. Attentional set shifting has been observed in humans and animals, including primates and rodents [104]. In rodents, the Birrell–Brown attentional set-shifting task (ASST) mimics the learning set required for successful performance of the Wisconsin Card Sorting Task (WCST), used to assess cognitive inflexibility in several neurological and psychiatric disorders [105].

The Birrell–Brown task consists of two stages that assess distinct facets of cognitive flexibility: the reversal (intra-dimensional shifting) stage and the extra-dimensional shift stage. During the reversal stage, previously irrelevant stimuli within the same dimension (e.g., odor cues) become relevant. Subsequently, attentional set-shifting is assessed during the extra-dimensional shift stage, in which the previously irrelevant dimension (e.g., texture) becomes the new focus of attention [106]. This task is a robust and replicable measure of the cognitive processes underlying attentional set-shifting behavior. Its main limitation is that deficits may reflect altered motivation, reward sensitivity, or odor discrimination rather than impaired cognitive flexibility per se.

An alternative that incorporates elements of the ASST and also measures higher-order repetitive behaviors is the Water T-Maze [107]. This assay is a sensitive, cost-effective, and less time-intensive alternative to dry-land foraging tasks. The maze is a versatile tool for evaluating spatial working memory, reversal learning (intra-dimensional shifting), and attentional (extra-dimensional) set shifting. The first two stages rely on a spatial cue; the escape platform is submerged at the end of the right or left arm during the initial stage (Spatial Discrimination), and its location is reversed after animals reach a learning criterion (Reversal Learning [82,108]). The final phase (ASST) is contingent on a previously irrelevant visual cue (e.g., a floating object or light), requiring an extra-dimensional shift [80]. As with the MWM, performance in the Water T-maze may be confounded by stress sensitivity, visual or motor abilities.

### 4.3. Executive Function and Problem Solving

Several tasks have been designed to study different aspects of executive function in rodents. The puzzle box task, created for rats and adapted for mice, is a good example of a task that assesses problem-solving aspects of executive control. Designed to mimic mental rotation and persistence tasks in humans, the puzzle box task requires planning, inhibition of ineffective strategies, and updating behavior based on prior experience, all core components of executive function [109].

In this task, mice complete escape challenges by moving from an open, brightly lit area to a dark, enclosed space. The difficulty of these escape tasks increases progressively across trials, all within a limited timeframe [110]. The animals’ motivation for “solving” the puzzle box stems from their innate tendency to avoid open spaces and seek shelter, as well as their natural inclination to explore their surroundings. These inherent drives result in concise testing procedures that require no extensive training or additional reinforcement [111]. The puzzle box engages a broad range of brain regions and demonstrates strong correlations with outcomes of other cognitive-behavioral tests [112]. However, it should be kept in mind that performance in the puzzle box task is limited by its sensitivity to anxiety, neophobia, and sensorimotor function, making it difficult to attribute poor performance exclusively to executive dysfunction or problem-solving deficits.

### 4.4. Social Cognition

Social cognition is a multifaceted process that involves interpreting social signals, intentions, and emotional states, as well as selecting appropriate responses [113]. This process includes elements such as reward-seeking, motivation, understanding of others, and the ability to adapt one’s social behavior.

A valuable test for assessing social cognition in rodents is the three-chamber social preference assay. In this test, the animal’s preference for interacting with a social stimulus (e.g., an unfamiliar mouse) over a non-social stimulus is evaluated [114]. A related task performed in the three-chamber apparatus is the social recognition assay, which requires social memory and discrimination, or the ability to remember a specific social stimulus and distinguish among conspecifics [115].

In addition to preference and recognition, nonviolent social interactions are an inherent trait among rodents. Rodents exhibit distinct “pro-social” behaviors, such as crawling under one another, allogrooming, huddling, following, and sniffing [114]. These “pro-social” behaviors have been observed in both male and female mice, across juvenile and adult stages, as exemplified by the Social Interaction Test (SIT) [115]. Although this task can be viewed as measuring purely social behaviors, some regard it as a cognitive assessment of the capacity to predict and adapt to uncertainty [61]. A general limitation of social behavioral assays in rodents is that performance reflects a composite of multiple interacting factors, including sensory cues (particularly olfactory processing), motivational state, anxiety, and social context, making it difficult to isolate specific social or cognitive components [62].

## 5. The Roles of Astrocytes in Cognition: Mechanistic Perspectives

Defining the role of astrocytes in cognition requires mechanistic approaches that move beyond association toward causal manipulation. As outlined above, rodent models allow for selective perturbation of astrocyte number, physiology, and intracellular signaling, enabling direct evaluation of how specific astrocytic processes shape cognitive performance. In the following sections, we examine evidence from interventions targeting global and experience-dependent astrocyte modulation; calcium signaling and homeostasis; gliotransmission and synaptic regulation; and, finally, inflammatory reactivity. Together, these findings delineate how distinct astrocytic mechanisms contribute to multidomain cognition (see Table 1 for a detailed breakdown of individual studies). Importantly, these mechanisms are not mutually exclusive; they interact dynamically to shape cognitive outcomes.

### 5.1. Global and Experience-Dependent Astrocyte Modulation

A global reduction in astrocyte function, especially in the prefrontal cortex (PFC), impairs several aspects of cognition. For example, prefrontal astrocytic ablation with the L-AAA toxin impairs recognition memory [61] and cognitive flexibility, including reversal learning and set-shifting [62,63]. Brain-wide inhibition of astrocytic metabolism via astrocyte-specific knockout of Carnitine Palmitoyltransferase-1A (CPT1A) also induces NOR deficits [64]. Inversely, chemogenetic astrocyte activation enhances recognition memory and cognitive flexibility [62].

Environmental factors, including social and physical manipulations, modulate astrocyte function and are closely linked to cognitive outcomes. Social isolation induces astrocytic atrophy and increased GFAP expression in the hippocampus, accompanied by NOR deficits, both of which can be reversed by anti-inflammatory treatment [65]. In contrast, positive environmental manipulations such as running and environmental enrichment enhance cognitive performance across multiple domains, including cognitive flexibility and memory, and are associated with changes in astrocyte morphology and complexity [66,67,68]. Notably, while aging and enrichment both increase astrocyte numbers, aging is characterized by a shift from Type 1 to the less complex Type 2 astrocytic phenotype; this shift is prevented by environmental enrichment [66,67]. Consistent with a causal role for astrocytes, astrocyte-specific overexpression of Glial Cell Line-Derived Neurotrophic Factor (GDNF) mitigates age-related cognitive decline [69]. Finally, cognitive demand itself, such as spatial learning in the MWM, can drive increases in astrocyte number, further supporting a bidirectional relationship between astrocytic plasticity and cognitive function [70,71]. Together, these findings highlight astrocytes as dynamic mediators of experience-dependent cognitive plasticity.

### 5.2. Calcium Signaling & Homeostasis

A fundamental approach to modulating astrocyte function involves altering intracellular calcium signaling and cell homeostasis. The disruption of astrocytic calcium release, e.g., by knockout of the intracellular calcium-release channel Inositol 1,4,5-trisphosphate receptor type 2 (IP3R2), reduced sociability and social novelty preference in the 3-chamber test [56], while leaving MWM spatial and reversal learning intact [72]. Similarly, reducing astrocyte calcium signaling by overexpressing the plasma membrane calcium-transporting ATPase 2 (PMCA2) impairs social interaction and preference; chemogenetic activation of astrocyte function is sufficient to restore these behavioral abnormalities [73]. In contrast, in pathological contexts, the disruption of astrocytic calcium signaling can have protective effects. For example, astrocyte-specific knockout of the calcium-dependent phosphatase subunit Calcineurin B1 (CaNB1) attenuates neuroinflammation and reactive gliosis, reduces pro-inflammatory cytokine release, and rescues recognition memory deficits in an Alzheimer’s disease model [74]. Together, these findings indicate that the cognitive impact of astrocytic calcium signaling depends not only on the specific pathway involved, but also on the physiological versus pathological context in which it is manipulated.

### 5.3. Gliotransmission & Synaptic Regulation

A related mechanism by which astrocytes influence cognitive function is through the regulation of synaptic transmission and plasticity, gliotransmitter release, and metabolic support of neural circuits. Blocking gliotransmission by reducing vesicular exocytosis in astrocytes, e.g., via astrocyte-specific expression of a dominant-negative SNARE (dnSNARE) domain [116,117] or intra-PFC tetanus neurotoxin (TeNT) administration, impairs spatial and recognition memory while leaving other cognitive domains intact. In parallel, these manipulations disrupt network-level oscillatory activity, as evidenced by desynchronization of hippocampal–prefrontal theta rhythms and reduced cortical gamma power [52,55]. Similarly, disrupting astrocytic network coupling via gap junction proteins, such as connexin 43 or connexin 36, impairs LTP and memory consolidation, although overexpression of astrocytic connexins has also been associated with memory decline [118]. Notably, the cognitive effects of gliotransmission are context-dependent: while its inhibition typically impairs cognitive performance, it can also confer protection under specific conditions. For example, inhibition of gliotransmitter release in dnSNARE mice attenuates sleep deprivation–induced cognitive deficits in the NOR task, an effect attributed to reduced extracellular adenosine signaling via A1 receptors [88].

Several studies indicate that astrocytes influence cognitive function in part by regulating inhibitory tone. Astrocyte-specific deletion of *Bmal1*, a gene involved in circadian clock regulation, reduces astrocytic GABA uptake, thereby increasing extracellular GABA and impairing recognition memory. Supporting the role of GABA control of memory processes, the authors found that pharmacological blockade of GABA-A receptors with pentylenetetrazole (PTZ) rescues the NOR deficit [75]. Consistent with this, astrocytes can also modulate inhibitory signaling by releasing trophic factors: astrocyte-derived brain-derived neurotrophic factor (BDNF), released in response to dexmedetomidine, suppresses excessive neuronal GABA-A receptor expression and protects against anesthesia-induced deficits in recognition and problem-solving [76].

Beyond directly regulating inhibitory tone, astrocytes further support cognitive function through metabolic and structural coupling with neurons. For example, astrocyte-specific deletion of apolipoprotein E (ApoE) disrupts cholesterol trafficking to neurons, leading to impaired synaptic maintenance and plasticity under baseline conditions and deficient circuit remodeling following traumatic brain injury, resulting in spatial memory deficits in the MWM and impaired cognitive recovery [87].

As described above, a critical aspect of astrocyte function is the clearance and metabolism of glutamate. Generally, manipulations that reduce glutamate reuptake or increase astrocytic glutamate release impair cognitive function. For example, pharmacological inhibition of glutamate uptake with the selective GLT-1 inhibitor dihydrokainic acid (DHK) significantly disrupts NOR [77]. In a rat model of depression (i.e., the Flinders Sensitive Line (FSL)), downregulation of the GLAST transporter and D-serine were correlated with cognitive impairments in NOR, but not with behavioral measures of anxiety or depression [78]. Similarly, genetically induced downregulation of GLAST leads to reduced social preference as well as social recognition, although social interaction remains unaffected [79]. Together, these findings indicate that astrocytic regulation of glutamate homeostasis is critical for multiple cognitive domains, with distinct aspects of behavior differentially sensitive to disruptions in glutamate clearance.

Another finding supporting the detrimental impact of elevated astrocytic glutamate release on cognition comes from studies of *Glud1*-deficient mice. *Glud1* (GDH; see Section 2) deletion in the CNS led to excess glutamate levels in the hippocampus and mPFC, impaired NOR performance, and reversal and attentional set-shifting in the water T-maze [80,81,82]. However, as these manipulations are not astrocyte-specific, the observed effects may reflect contributions from both neuronal and glial GDH deficiency. Recently, we have shown that an astrocyte-specific deletion of *Glud1* induces deficits in water T-maze reversal (Andrianov and Gaisler-Salomon, in preparation). These findings provide preliminary evidence that astrocytic GDH-dependent glutamate metabolism may contribute to cognitive flexibility and memory processes; however, given GDH’s broad metabolic role, the behavioral effects may also arise from more widespread alterations in cellular metabolism and network function. More targeted disruption of astrocytic glutamate release mechanisms, e.g., deletion of the astrocyte-specific cystine/glutamate antiporter system (System xc−, Sxc), which exchanges extracellular cystine for intracellular glutamate, did not affect NOR performance but did lead to perseverative behavior in an attentional set-shifting T-maze task in rats [83]. This indicates that astrocytic manipulations that increase extracellular glutamate may specifically hinder performance in tasks that require cognitive flexibility.

Given the prominent role of the NMDA receptor in cognitive processes, a relevant question is whether astrocytes can modulate its function, thereby affecting memory and attentional capacities. Several studies support this possibility. For example, mice with genetically induced deletion of Bestrophin-1 (Best1), a calcium-activated anion channel with significant permeability to glutamate and GABA, show reduced NMDAR tone and impaired reversal learning in the MWM. This deficit is rescued by astrocyte-specific re-expression of *Best1* or by supplementation with the NMDA receptor co-agonist D-serine during the initial learning phase [84]. Consistent with a role for astrocyte-derived D-serine in modulating NMDAR-dependent behaviors, social deficits induced by astrocyte-specific expression of mutant Disrupted-In-Schizophrenia 1 (DISC1) are similarly reversed by D-serine [85]. In addition, knockdown of the GluN2A subunit of NMDA receptors specifically in astrocytes exacerbated NOR memory deficits induced by amyloid-beta oligomers [86], providing further support for astrocytic contributions to NMDA receptor-mediated signaling and cognition.

### 5.4. Inflammation & Reactivity

The transformation of astrocytes into reactive states is a key driver of cognitive change, particularly under inflammatory conditions. Across multiple models, including LPS-induced inflammation, ischemic stroke, post-operative stress, and pharmacological models, astrocyte activation is consistently associated with upregulated expression of GFAP and pro-inflammatory cytokines (TNF-α, IL-1, IL-6), alongside impairments in spatial and recognition memory, effects that can often be mitigated by suppressing astrocyte reactivity or inflammatory pathways [86,88,89,90,91]. Mechanistically, these effects are mediated in part by astrocytic Toll-like receptor 4 (TLR4) signaling and downstream pathways such as NF-κB, as well as broader immune-modulatory processes that shape the local inflammatory microenvironment. In addition, activation of the astrocytic NLRP3 inflammasome has emerged as a central contributor to inflammation-associated cognitive dysfunction across conditions, including chronic kidney disease and chemotherapy exposure [92,93]. Importantly, astrocyte reactivity is not uniformly detrimental: specific stress-response pathways can exert protective effects, as disruption of Transient Receptor Potential Ankyrin 1 (TRPA1) or Protein Kinase RNA-Like Endoplasmic Reticulum Kinase (PERK) signaling exacerbates cognitive deficits and tissue damage in models of hypoperfusion, aging, and stroke [94,95]. Together, these findings highlight astrocyte reactivity as a context-dependent regulator of cognition, with both detrimental and protective roles mediated by distinct inflammatory pathways.

## 6. Summary and Conclusions

Astrocytes play an integral and dynamic role in cognitive function, engaging in reciprocal interactions with neuronal circuits and exhibiting marked plasticity in response to environmental demands. Across paradigms, alterations in astrocytic signaling, metabolic support, and reactivity consistently translate into measurable cognitive outcomes, although these effects are often domain-specific and, in some cases, divergent across tasks.

A key emerging principle is that astrocytic mechanisms do not exert uniform effects on cognition. While some perturbations lead to global impairments, others selectively disrupt specific domains such as cognitive flexibility or social behavior and may even enhance performance in parallel domains. This suggests that distinct astrocytic processes differentially modulate discrete cognitive functions, rather than contributing to cognition in a generalized manner.

Despite these advances, several critical gaps remain. First, the field continues to rely heavily on memory-based assays, particularly the NOR and MWM, which do not capture the full spectrum of cognitive functions affected by astrocytic dysfunction. Expanding the behavioral repertoire to include domains such as attention, executive function, and problem-solving will be essential for a more comprehensive understanding of astrocyte-dependent cognition.

Second, the contribution of astrocyte heterogeneity to cognition remains poorly understood. While broad classifications of reactive states have been described, the functional roles of region-specific and molecularly distinct astrocyte subpopulations are largely unresolved. Addressing this will require developing and applying tools with improved cellular and spatial specificity.

Third, a major limitation of the current literature is the restricted consideration of sex as a biological variable. Many studies of astrocyte function have been conducted in a single sex, often males, and direct comparisons between males and females are rare. Given evidence for sex-dependent differences in astrocyte biology, this represents a significant gap. Systematic inclusion of both sexes and explicit testing for sex effects will be essential to determine whether astrocytic mechanisms of cognitive regulation generalize across sexes or contribute to sex-specific phenotypes.

Finally, a key conceptual challenge is mapping specific astrocytic mechanisms to distinct cognitive domains. We propose that processes such as gliotransmission, metabolic support, and neuroinflammatory signaling contribute differentially to memory, cognitive flexibility, and social behavior, respectively. Testing this hypothesis will require integrative approaches that combine precise astrocyte-targeted manipulations with multidimensional behavioral phenotyping.

To bridge the translational gap, future research should move beyond standard memory assays and incorporate high-throughput, ethologically relevant behavioral batteries. In parallel, advances in targeting defined astrocyte subpopulations, including the use of cell-type-specific promoters and single-nucleus sequencing approaches, will be critical for establishing causal links between astrocyte diversity and cognitive function.

Understanding how astrocytes regulate specific cognitive domains provides crucial insights into their contribution to brain pathology. Furthermore, because astrocytic dysfunction is a core feature of many neurological and psychiatric disorders, these astrocytic pathways represent highly promising targets for the development of disease-modifying therapeutics. For example, modulating IP3R2 receptors, PMCA2 calcium pumps, or Gq pathways has been shown to restore synaptic plasticity, rescue social cognition, and improve cognitive flexibility. Furthermore, targeting gliotransmission, such as restoring NMDA receptor tone via astrocyte-derived D-serine to render memories flexible, utilizing glial glutamate clearance activators, and adenosine A2a receptor agonists can successfully bypass astrocytic signaling deficits. Finally, astrocytic inflammatory pathways represent viable targets for therapeutic development. Modulating these pathways or suppressing reactive astrogliosis could help halt cognitive decline associated with pathology.

## Data Availability

No new data were created or analyzed in this study. Data sharing is not applicable to this article.
